# Supplementary material for: Generation and characterization of human iPSC-derived NPC1I1061T/I10161T i3Neurons as a model for NPC1 disease
Source: bioRxiv. 2026 Feb 13:2026.02.11.705111. Preprint. [Version 1] doi: 10.64898/2026.02.11.705111 (PMC12918829; doi:10.64898/2026.02.11.705111)
Supplement: Supplement 1 [file media-1.pdf]

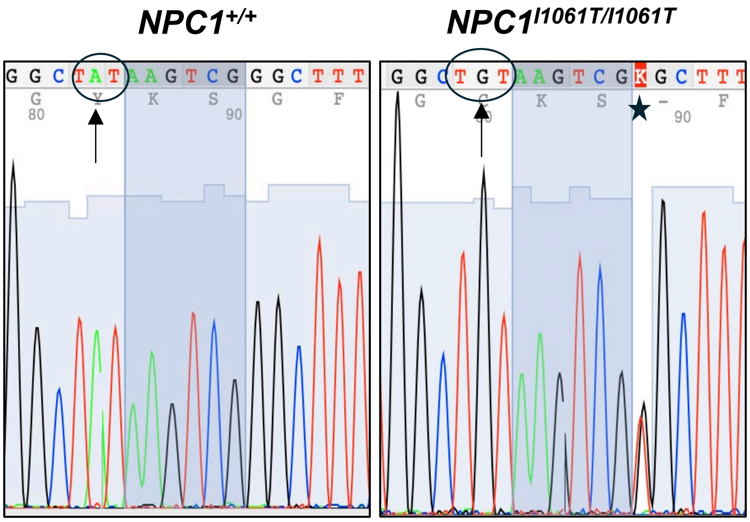

Supplementary Figure 1. Reverse Sanger sequencing analysis of the genomic DNA from *NPC1*$^{+/+}$ and *NPC1*$^{I1061T/I1061T}$ cells. In this direction, the A>G mutation (arrows), resulting in the isoleucine to threonine change is confirmed, along with the silent mutation (star).

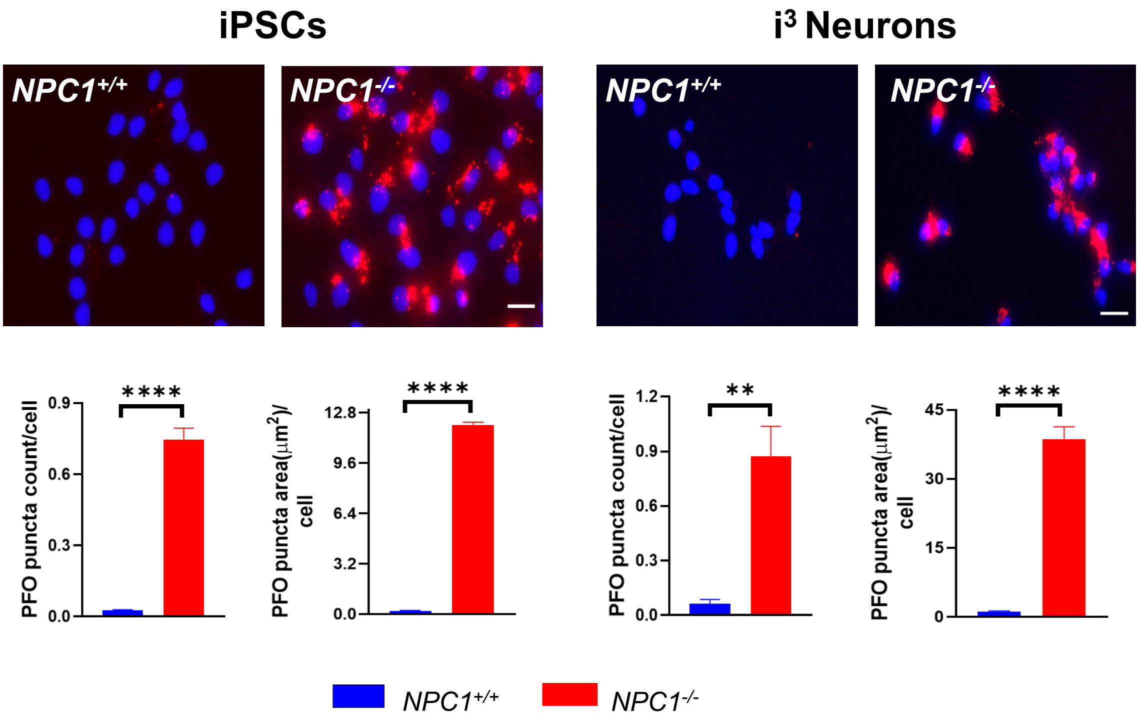

Supplementary Figure 2. Analysis of cholesterol accumulation in lysosomes of *NPC1*$^{+/+}$ and *NPC1*$^{-/-}$ iPSCs and i^{3}Neurons using PFO-647 staining (red). The upper panels show strong PFO staining in both *NPC1*$^{-/-}$ iPSCs and i^{3}Neurons. The lower panel shows the results of the quantification by ImageJ. The size of puncta (μm^2) per cell increased by 54-fold ($p<0.0001$) and 34-fold ($p<0.0001$) in *NPC1*$^{-/-}$ iPSCs and i^{3}Neurons, respectively, compared to *NPC1*$^{+/+}$ cells. Similarly, the number of PFO puncta per cell was 28-fold higher in *NPC1*$^{-/-}$ iPSCs ($p<0.0001$) and 14-fold higher in *NPC1*$^{-/-}$ i^{3}Neurons ($p<0.01$) when compared to *NPC1*$^{+/+}$ iPSCs and i^{3}Neurons, respectively. The nuclei were counterstained with DAPI (blue). 100 cells were counted per cell line and data obtained from three independent experiments (n=3). Scale bar, 20 μm.**$p<0.01$ and ****$p<0.0001$ using unpaired t-test when comparing two independent samples**.**

Salhotra et al. Supplementary Figure 2

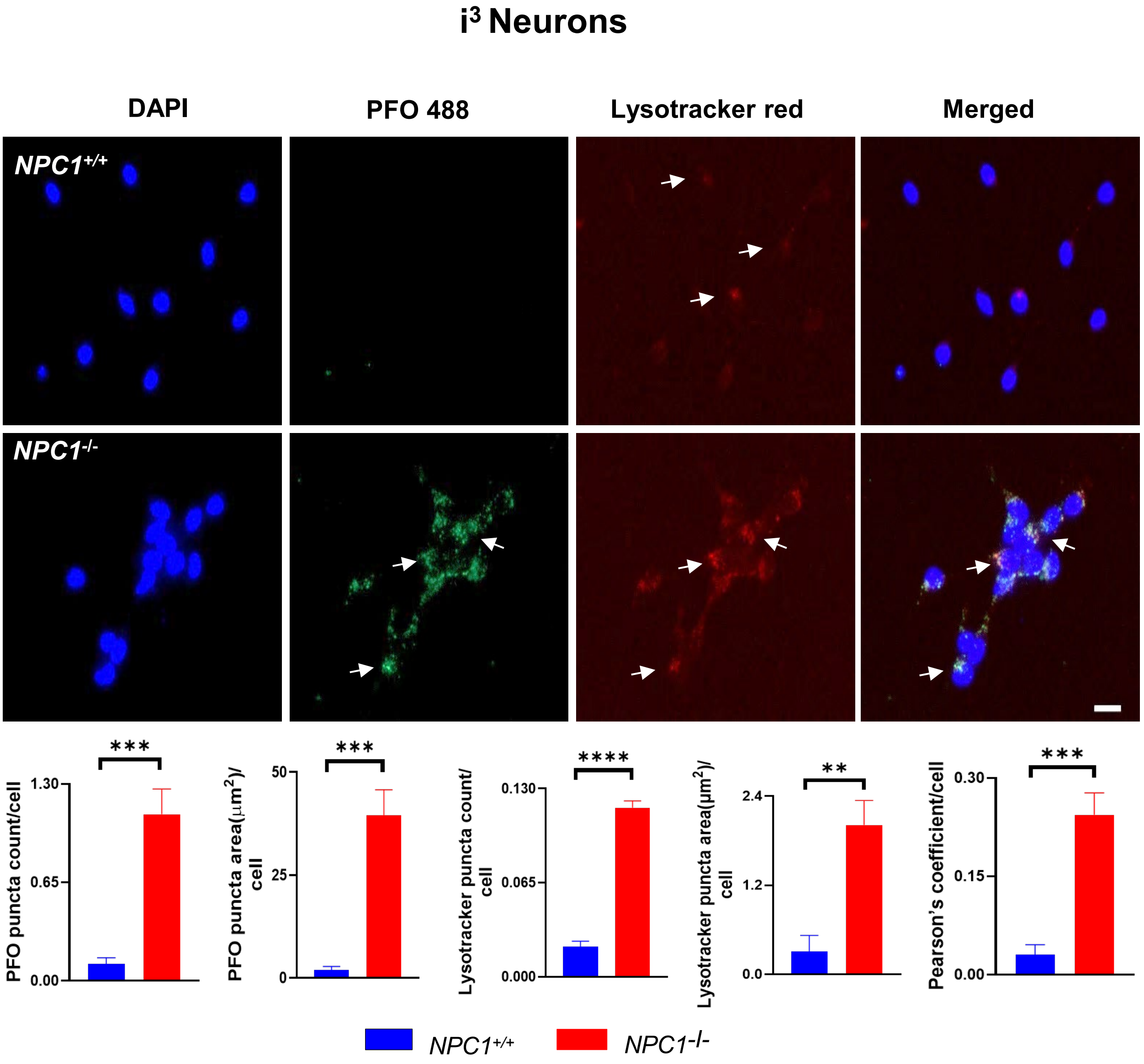

Supplementary Figure 3. Analysis of *NPC1*$^{+/+}$ and *NPC1*$^{-/-}$ i^{3}Neurons for cholesterol staining with PFO-488. Unesterified cholesterol (arrows) was found to strongly co-stain with lysotracker red, indicating the accumulation in lysosomes in *NPC1*$^{-/-}$ neurons. PFO puncta area increased by 20 fold ($p<0.001$) and PFO puncta count increased by 9.8 fold ($p<0.001$) in *NPC1*$^{-/-}$ i^{3}Neurons compared to *NPC1*$^{+/+}$. Similarly, lysotracker red puncta count and puncta area per cell increased by 5.63 fold ($p<0.0001$) and 6.5 fold ($p<0.01$), respectively in *NPC1*$^{-/-}$ i^{3}Neurons. Pearson's coefficient was found to be 8.1 fold higher in *NPC1*$^{-/-}$ i^{3}Neurons ($p<0.001$). The nuclei were counterstained with DAPI (blue). 100 cells were counted per cell line and data obtained from three independent experiments (n=3). Scale bar, 20 μm.**$p<0.01$, ***$p<0.001$ and ****$p<0.0001$ using unpaired t-test when comparing two independent samples.

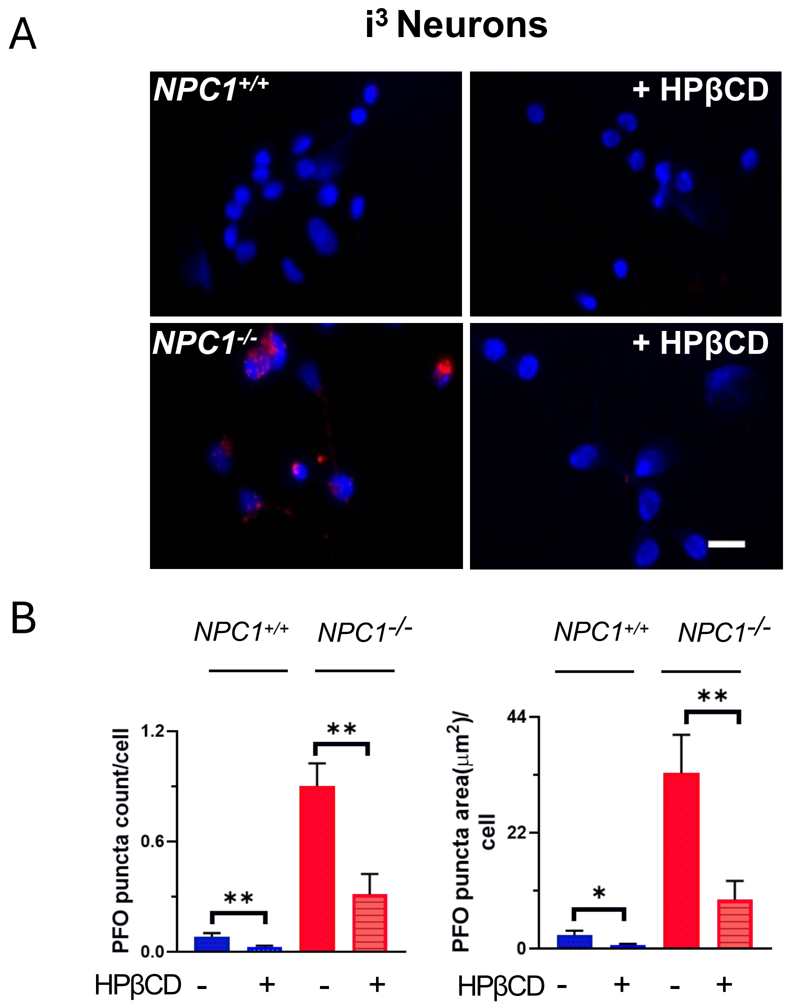

Supplementary Figure 4. Demonstration of the reversal of PFO staining in *NPC1*$^{-/-}$ i^{3}Neurons with 2-Hydroxypropyl-β-cyclodextrin (HPβCD) treatment. (**A**) Fluorescence microscopy of *NPC1*$^{+/+}$ and *NPC1*$^{-/-}$ i^{3}Neurons showing PFO staining (red) in the mutant cells that is reduced when treated with HPβCD. (**B**) Quantification of the staining using ImageJ. PFO puncta count was reduced 2.9-fold ($p < 0.01$) and puncta area was reduced 3.5-fold ($p < 0.01$) after HPβCD treatment in *NPC1*$^{-/-}$ i^{3}Neurons. DAPI (blue) was used to counterstain the nuclei. 100 cells were counted per cell line and data obtained from three independent experiments (n=3). Scale bar, 20 μm.*p<0.05, **p<0.01 using unpaired t-test when comparing two independent samples.
